# Serum potassium and high-altitude coronary microvascular disease: a propensity score matched analysis revealing a linear dose-response relationship

**DOI:** 10.3389/fphar.2026.1826893

**Published:** 2026-06-29

**Authors:** Yu Chen, Ming Shen, Xin Zhang, Yong Ren, Wen-Tao Zhang, Juan Lv, Xi-yang Rao, Si-Jie Wang, Li-Sha Wang, Zhu Yan, Chun-ping Bao, Rui-Xi Zi, Gong-hao He, Li-Xia Yang, Yan-Kun Shi

**Affiliations:** 1 Department of Cardiology, 920th Hospital of Joint Logistics Support Force of PLA, Kunming, China; 2 Department of Cardiology, Yueyang Hospital of Integrated Traditional Chinese and Western Medicine, Shanghai University of Traditional Chinese Medicine, Shanghai, China; 3 Department of Pulmonary and Critical Care Medicine, 920th Hospital of Joint Logistics Support Force of PLA, Kunming, China; 4 Department of Clinical Medical College, Dali University, Dali, China; 5 Department of Clinical Pharmacy, 920th Hospital of Joint Logistics Support Force of PLA, Kunming, China

**Keywords:** coronary microvascular disease, dose-response relationship, high altitude, propensity score matching, serum potassium

## Abstract

**Background:**

High-altitude exposure precipitates coronary microvascular disease (CMVD), a pathology linked to hypoxia-induced ion channel dysfunction. However, prior observational evidence connecting serum potassium to CMVD resilience is limited by residual confounding, and the precise dose-response trajectory remains unmapped. This study utilized Propensity Score Matching (PSM) to rigorously isolate this association and characterize the dose-response trajectory across the observed physiological range.

**Methods:**

We conducted a nested case-control analysis within a high-altitude training cohort. To minimize selection bias, 1:1 PSM was employed to strictly balance covariates—including age, blood pressure, and autonomic nervous regulation—between CMVD cases and controls. The final analytic sample comprised 462 participants (231 pairs). We applied conditional logistic regression and dose-response modeling to evaluate the association between pre-high-altitude serum potassium and CMVD risk.

**Results:**

Post-matching analysis confirmed that higher baseline serum potassium was independently associated with a significantly reduced CMVD likelihood. The adjusted odds ratio was 0.18 (95% CI: 0.08–0.42, *P* <0 .001) per 1 mmol/L increase, translating to a clinically actionable 16% risk reduction for every 0.1 mmol/L increment.

**Conclusion:**

Higher physiological serum potassium is robustly and linearly associated with reduced likelihood of CMVD under high-altitude hypoxic conditions. These association-based findings suggest the potential value of further investigating whether maintaining higher physiological potassium levels before high-altitude deployment may reduce CMVD risk, moving beyond merely screening for hypokalemia.

## 1 Introduction

Hypoxia-induced microvascular dysfunction contributes significantly to myocardial ischemia, posing a particular risk to individuals exposed to the physiological stress of high-altitude environments (Scarica et al., 2025; Lanza et al., 2010). Hypoxia-induced alterations in ion channel function, specifically involving ATP-sensitive potassium (K_ATP_) channels, have been implicated in the pathogenesis of microvascular dysfunction (Holdsworth et al., 2017; Quayle et al., 2006; Rehan et al., 2023).

In our previous case-control investigation involving 1,175 high-altitude training participants (Chen et al., 2025), multivariable unconditional logistic regression revealed a significant inverse association between pre-high-altitude serum potassium and CMVD risk (OR = 0.26; 95% CI: 0.14–0.47), and further identified potential effect modification by BMI and smoking status. While that study provided foundational evidence that higher serum potassium levels were associated with lower CMVD odds, it carried two methodological limitations that constrain the interpretability of this association. First, significant baseline imbalances in age, BMI, and systolic blood pressure existed between the CMVD and non-CMVD groups prior to analysis, rendering the observed association susceptible to residual confounding that standard multivariable regression cannot fully eliminate. Second, the exposure variable was treated as a categorical predictor in subgroup analyses, leaving the precise, continuous shape of the potassium–CMVD association entirely uncharacterized—specifically, whether the association follows a linear gradient across the physiological range or exhibits a non-linear threshold pattern.

The present study was designed to address both of these limitations and thereby provide a distinct, incremental contribution. We employed Propensity Score Matching (PSM) (Austin, 2011) to construct a covariate-balanced analytic sample, thereby reducing the influence of the confounders identified as imbalanced in our prior work. Crucially, we further applied continuous dose-response modeling to characterize the specific shape of the potassium–CMVD association for the first time. Together, these methodological advances move beyond the binary question posed by our earlier study—“is lower potassium associated with higher CMVD risk?”—to address a more granular and clinically relevant question: “across what range, and to what degree, is serum potassium level associated with CMVD risk?” This Brief Research Report thus represents a planned methodological extension of our prior findings, rather than a replication, aiming to provide a more bias-adjusted and quantitatively precise characterization of this association in personnel preparing for high-altitude deployment.

## 2 Methods

### 2.1 Study design and participants

This investigation was structured as a PSM case-control analysis embedded within a previously established cohort of participants engaged in high-altitude training (Chen et al., 2025). The detailed recruitment process, inclusion/exclusion criteria, and ethical approvals have been reported previously. Briefly, data were collected from the 920th Hospital of the Joint Logistics Support Force between January and June 2022. In the original cohort, participants with pneumonia, hypertension, or structural heart disease were excluded. For the current analysis, we focused on establishing a balanced comparison to minimize selection bias. The final analytic sample consisted of 462 participants (231 CMVD cases and 231 controls) derived through PSM from the original cohort (Supplementary Figure S1).

### 2.2 Clinical measurements and CMVD diagnosis

Clinical measurements, including demographics, smoking status, and blood biochemistry (including serum potassium), were obtained using standard protocols as previously described (Chen et al., 2025). To minimize dietary variability, subjects consumed a standardized meal (bread and milk) the night before blood sampling. Specifically, fasting venous blood samples were collected in the morning to measure serum potassium levels using an automated biochemical analyzer.

The diagnosis of CMVD followed the same rigorous two-step criteria used in our primary study: (1) Electrocardiogram (ECG) evidence of myocardial ischemia (ST-segment depression >0.5 mm or T-wave inversion ≥1 mm in contiguous leads), followed by (2) confirmation via Transthoracic Doppler Echocardiography (TDE) showing a Coronary Flow Reserve (CFR) ≤ 2.3. Full procedural details, including equipment specifications and offline analytical methods, are provided in our primary publication (Chen et al., 2025).

### 2.3 Statistical analysis

To mitigate confounding inherent in observational designs, we performed PSM using the R package MatchIt (v4.5.5). Propensity scores were estimated using a logistic regression model including age, sex, BMI, SBP, LDL-C, smoking status, and SCOPA-AUT scores (to account for autonomic nervous system regulation) as covariates. A 1:1 nearest neighbor matching algorithm without replacement was applied. To ensure close matching, a caliper width of 0.2 standard deviations of the logit of the propensity score was used. Covariate balance was assessed using the Absolute Standardized Mean Difference (ASMD), with values < 0.1 considered indicative of good balance. Missing data were handled using complete-case analysis (5% missingness).

Following PSM, doubly robust estimation was employed to estimate the association between serum potassium and CMVD. Specifically, we used multivariate conditional logistic regression models adjusting for any residual imbalances in covariates after matching. To examine potential non-linear relationships, we compared the linear model with a quadratic model using the Likelihood Ratio Test. The addition of a quadratic term did not significantly improve model fit (*P* = 0.58). Consequently, a linear logistic regression model was adopted. All analyses were performed using R software (version 4.2.0), with a two-sided P-value <0.05 considered statistically significant.

## 3 Results

### 3.1 Participant characteristics and PSM balance

**FIGURE 1:**
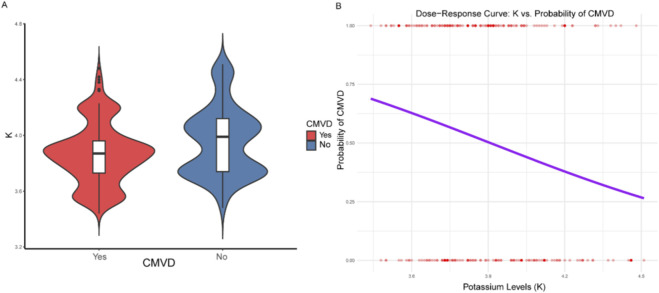
Violin Plot Comparing Potassium (K) Levels in CMVD Cases and Controls and Dose-Response Curve Between Potassium Levels and the Probability of CMVD. **(A)** The violin plot illustrates the distribution of potassium levels in individuals diagnosed with Coronary Microvascular Disease (CMVD) compared to controls. Red violins represent the CMVD group, while blue violins represent the control group. The width of the violin indicates the density of data points at different potassium levels. Boxplots within the violins show the median, interquartile range (IQR), and potential outliers. **(B)** The dose-response curve displays the predicted probability of developing CMVD based on a logistic regression model. The purple line shows a negative association between higher potassium levels and the risk of CMVD, with the X-axis representing potassium concentrations (K, mmol/L) and the Y-axis depicting the predicted probability of CMVD. The scatter plot highlights individual data points across potassium levels, color-coded by CMVD status.

The initial cohort included 2,855 participants, from whom 462 participants (231 CMVD cases and 231 matched controls) were selected through 1:1 PSM (see Supplementary Figure S1 for the detailed participant selection and matching flowchart). Significant baseline imbalances were observed between the CMVD and control groups regarding age, BMI, and SBP prior to matching (Table 1). Following PSM, all covariates were well balanced, as evidenced by ASMD values reduced to <0.1 across all variables (Table 1; Supplementary Table S1). This balance was further confirmed visually by the Love Plot (Supplementary Figure S2), which illustrates the substantial reduction in standardized mean differences for all covariates after matching. Baseline characteristics showed no significant differences between the groups after matching. However, the exposure variable, serum potassium, remained significantly different between groups (pre-high-altitude: 3.87 vs. 3.99 mmol/L, P = 0.001; Figure 1A), consistent with it being the primary exposure under investigation rather than a matching covariate.

**TABLE 1:** Baseline characteristics for cases and controls of CMVD before and after PSM.

Variables	Before PSM (n = 2,855)			After PSM (n = 462)		
	Cases (n = 235)	Controls (n = 2,620)	*P* value	Cases (n = 231)	Controls (n = 231)	*P* value
Age (years)	24.0 [22.0; 29.0]	24.0 [22.0; 26.0]	<0.001	24.0 [22.0; 29.0]	24.0 [22.0; 27.0]	0.764
BMI (kg/m^2^)	22.6 [20.9; 24.3]	22.1 [20.8; 23.7]	0.029	22.6 [20.9; 24.3]	22.5 [21.0; 24.2]	0.757
Sex	​	​	0.028	​	​	1.000
Female	8 (3.40%)	37 (1.41%)	​	6 (2.60%)	5 (2.16%)	​
Male	227 (96.6%)	2,583 (98.6%)	​	225 (97.4%)	226 (97.8%)	​
SBP (mmHg)	119 [110; 125]	117 [110; 120]	0.001	120 [110; 125]	120 [112; 124]	0.783
OS (%)	93.0 [92.0; 95.5]	93.0 [92.0; 95.0]	0.051	93.0 [92.0; 96.0]	93.0 [92.0; 95.0]	0.174
K, pre-high-altitude (mmol/L)	3.87 [3.73; 3.96]	3.93 [3.74; 4.12]	<0.001	3.87 [3.73; 3.96]	3.99 [3.74; 4.12]	0.001
K, post-high-altitude (mmol/L)	3.70 [3.34; 4.12]	3.81 [3.43; 4.20]	0.010	3.68 [3.33; 4.12]	3.83 [3.42; 4.26]	0.025
LDL-C, pre-high-altitude (mmol/L)	2.90 (0.43)	2.91 (0.50)	0.767	2.89 (0.44)	2.90 (0.47)	0.865
Smoking status	​	​	0.291	​	​	0.926
Never	106 (45.1%)	1,246 (47.6%)	​	103 (44.6%)	107 (46.3%)	​
Quitted	14 (5.96%)	211 (8.05%)	​	14 (6.06%)	13 (5.63%)	​
Smoking	115 (48.9%)	1,163 (44.4%)	​	114 (49.4%)	111 (48.1%)	​
SCOPA-AUT score (post-high altitude)	5.00 [3.00; 9.00]	6.00 [3.00; 10.0]	0.693	5.00 [3.00; 9.00]	6.00 [3.00; 9.00]	0.433

Data are presented as median [interquartile range] for non-normally distributed continuous variables, mean (standard deviation) for normally distributed continuous variables. Statistical comparisons were performed using the Mann–Whitney U test, independent samples t-test, or χ^2^ test, as appropriate. High altitude was defined as an altitude ≥2,500 m. BMI: body mass index; OS: oxygen saturation; K: serum potassium; LDL: Low-Density Lipoprotein Cholesterol; SBP: systolic blood pressure; SCOPA-AUT: the Scale for Outcomes in Parkinson’s Disease for Autonomic Symptoms; PSM: propensity matching score.

### 3.2 Association between potassium and CMVD likelihood

In the propensity score-matched cohort, conditional logistic regression analysis demonstrated that higher pre-high-altitude serum potassium levels were independently associated with a significantly lower likelihood of CMVD (Table 2). Dose-response analysis characterized this relationship as a linear inverse association (*P* for non-linearity = 0.58; Figure 1). Based on this linear model, the adjusted odds ratio (OR) was 0.18 (95% CI: 0.08–0.42, *P* < 0.001). This translates to an OR of approximately 0.84 (95% CI: 0.77–0.92) for every 0.1 mmol/L increase, suggesting a 16% reduction in the odds of CMVD for each 0.1 mmol/L increment.

**TABLE 2:** Univariate and multivariate conditional binary logistic regression analyses for the relationship between CMVD following high-altitude exposure and clinical characteristics.

Variables	Univariate		Multivariate	
	OR (95% CI)	P	OR (95% CI)	P value
K (pre-high altitude) (mmol/L)	0.18 (0.08–0.42)	<0.001	0.18 (0.08–0.42)	<0.001
Age (years)	1.00 (0.97–1.04)	0.971	1.00 (0.96–1.04)	0.851
BMI (kg/m2)	1.00 (0.92–1.09)	0.968	1.01 (0.92–1.10)	0.878
SBP (mmHg)	1.00 (0.98–1.01)	0.644	1.00 (0.98–1.02)	0.894
Sex				
Male (reference)	—	—	—	—
Female	1.21 (0.36–4.01)	0.761	1.46 (0.39–5.45)	0.575
LDL-C (pre-high altitude) (mmol/L)	0.97 (0.64–1.45)	0.864	0.95 (0.62–1.44)	0.806
Smoking status				
Never (reference)	—	—	—	—
Quitted	1.12 (0.50–2.49)	0.784	1.35 (0.59–3.09)	0.482
Smoking	1.07 (0.73–1.55)	0.736	1.10 (0.74–1.62)	0.651
SCOPA-AUT score (post-high altitude)	0.99 (0.96–1.03)	0.690	0.99 (0.95–1.02)	0.510

The multivariable model is a conditional logistic regression performed within the propensity score-matched cohort, incorporating all listed covariates simultaneously regardless of their univariate significance (doubly robust estimation). This approach was pre-specified to adjust for any residual imbalance remaining after propensity score matching. OR: odds ratio; CI: confidence interval; K: serum potassium; BMI: body mass index; SBP: systolic blood pressure; LDL-C: low-density lipoprotein cholesterol; SCOPA-AUT: Scale for Outcomes in Parkinson’s Disease for Autonomic Symptoms.

### 3.3 Dose-response relationship analysis

To further characterize the association between serum potassium levels and the likelihood of CMVD, a dose-response analysis was performed using a binary logistic regression model with potassium entered as a continuous variable. The resulting probability curve (Figure 1B) illustrates a distinct inverse relationship. As baseline potassium concentrations prior to high-altitude exposure rose, the estimated likelihood of developing CMVD demonstrated a corresponding gradual decrease. This trend was consistent across the observed physiological range of potassium (approximately 3.5–4.5 mmol/L), reinforcing the findings from the multivariate analysis that higher potassium levels are associated with a lower likelihood of CMVD.

## 4 Discussion

The present study addressed two specific methodological limitations identified in our prior work (Chen et al., 2025), and the findings are consistent with both hypotheses articulated in the Introduction. First, following 1:1 propensity score matching that substantially reduced baseline covariate imbalances, the inverse association between pre-high-altitude serum potassium and CMVD risk was not only preserved but appeared more pronounced (OR = 0.18; 95% CI: 0.08–0.42) than the estimate derived from our unmatched cohort (OR = 0.26; 95% CI: 0.14–0.47), corroborating the hypothesis that the original estimate may have been attenuated by residual confounding. Second, continuous dose-response modeling confirmed that this inverse association follows a strictly linear pattern across the physiological potassium range, with no statistically significant evidence of non-linearity, validating the hypothesis that the relationship is graded rather than threshold-dependent. Taken together, these findings suggest that the inverse association between serum potassium and CMVD risk is unlikely to be an artifact of demographic imbalances between groups, and that it extends continuously across the physiological range rather than emerging only below a discrete cutoff—two observations that meaningfully refine the interpretation of our prior case-control evidence.

Although our clinical data cannot directly elucidate the underlying molecular pathways, several hypothesis-generating mechanisms may be considered. Potassium contributes to membrane electrochemical stability through the Na^+^/K^+^-ATPase pump and multiple K^+^ channel subtypes, not limited to K_ATP_ channels (Gao et al., 2021; Smith et al., 2021; Barreira et al., 2026; Zheng et al., 2019). However, because serum potassium levels in this study were largely within the physiological range of 3.5–4.5 mmol/L, only modest changes in endothelial or vascular smooth muscle membrane potential would be expected; thus, the observed inverse association should not be interpreted as evidence of a K_ATP_-specific causal mechanism or substantial potassium-induced hyperpolarization. Potassium may also influence endothelial homeostasis, including shear stress-related eNOS/NO signaling (Oberleithner et al., 2009; Bartosch et al., 2021), but this was not directly assessed. In addition, although high-altitude hypoxia may activate the Renin-Angiotensin-Aldosterone System (RAAS) (Kong et al., 2023; Palubiski et al., 2020), and high dietary potassium has been shown experimentally to suppress intrarenal renin and other RAAS components (Gonzalez et al., 2019; Vio et al., 2020), these findings derive from supraphysiological potassium interventions. Whether physiological variation in serum potassium can meaningfully modulate RAAS activity in humans at high altitude remains unknown and requires direct mechanistic validation.

The validation of our previous findings using PSM strengthens the rationale for further investigating potassium monitoring in this population. Moving beyond the screening approach suggested in our 2025 paper, the linear association observed here suggests the potential value of a more proactive “optimization” strategy. It suggests that, within the observed physiological range, higher potassium values were continuously associated with lower CMVD odds, with no evidence of a threshold below which the association disappears or an upper bound beyond which further benefit plateaus. While this linear pattern does not statistically identify a discrete optimal target range, it is consistent with the notion that maintaining potassium in the higher portion of the physiological range may be associated with lower CMVD likelihood—a hypothesis that prospective intervention studies would be required to evaluate.

## 5 Strengths and limitations

The primary strength is the application of PSM within a nested case-control framework, rigorously minimizing confounding bias. However, limitations exist. First, the 1:1 matching structure fixes outcome prevalence at 50%, meaning results represent odds ratios rather than absolute risks. Second, we relied on single baseline potassium measurements without longitudinal tracking. Third, including the post-exposure variable SCOPA-AUT in our propensity score model introduces a potential risk of post-exposure adjustment bias. Finally, the young male cohort limits generalizability to females and older populations.

## 6 Conclusion

Using a PSM-based nested case-control analysis, this study confirms that higher pre-high-altitude serum potassium is robustly associated with reduced CMVD risk. Uniquely, we identified a linear dose-response relationship, demonstrating that this inverse association extends continuously across the physiological range. Theoretically, this is consistent with potassium’s proposed role in vascular stability. Clinically, our findings suggest that the continuous, linear nature of this association across the observed physiological range—rather than a discrete threshold effect—warrants prospective investigation into whether broader attention to potassium status, beyond merely screening for hypokalemia, may be beneficial in high-altitude populations.

## Data Availability

The datasets generated and/or analyzed during the current study are not publicly available due to military secrecy but are available from the corresponding author on reasonable request. Requests to access these datasets should be directed to sykdoctor@outlook.com.
