# Supplementary material for: Serum potassium and high-altitude coronary microvascular disease: a propensity score matched analysis revealing a linear dose-response relationship
Source: Front Pharmacol. 2026 Jun 29;17:1826893. doi: 10.3389/fphar.2026.1826893 (PMC13357738; doi:10.3389/fphar.2026.1826893)
Supplement: Supplementary file 1 [file Supplementaryfile1.docx]

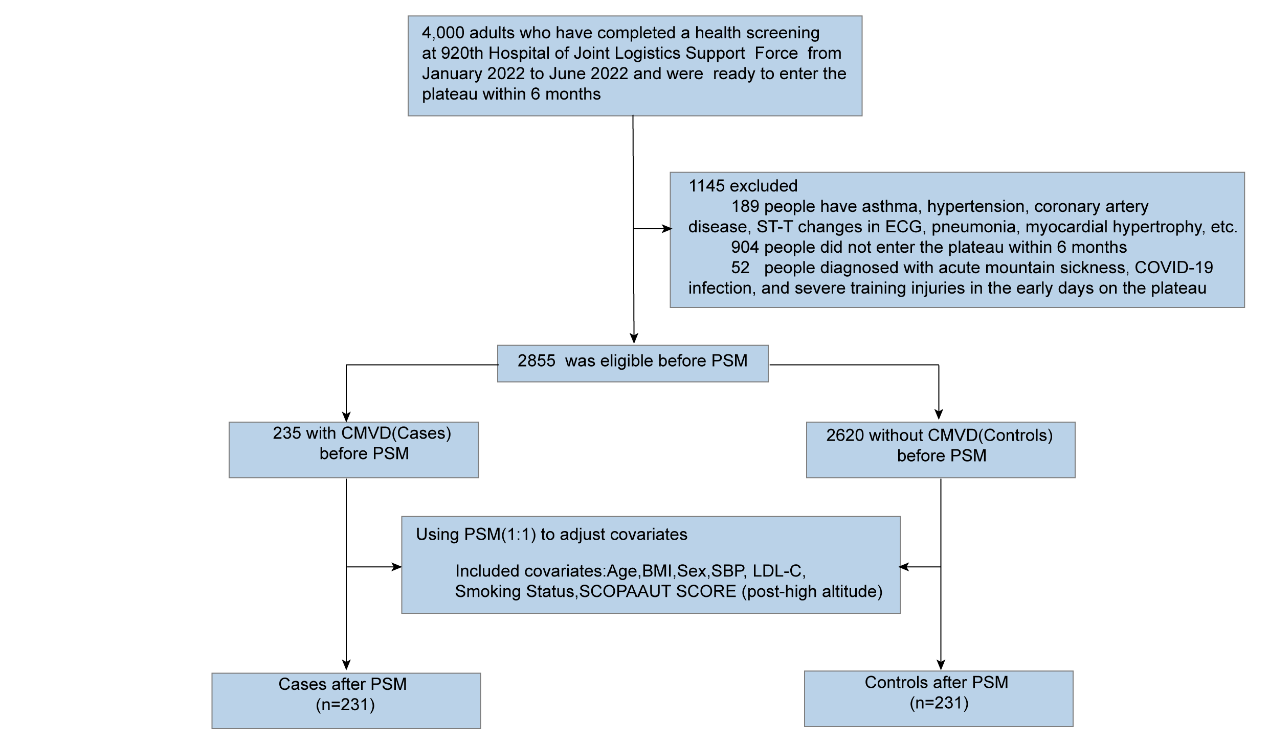


**Fig S1. Study Flowchart of Participant Selection and Propensity Score Matching (PSM) Process.**


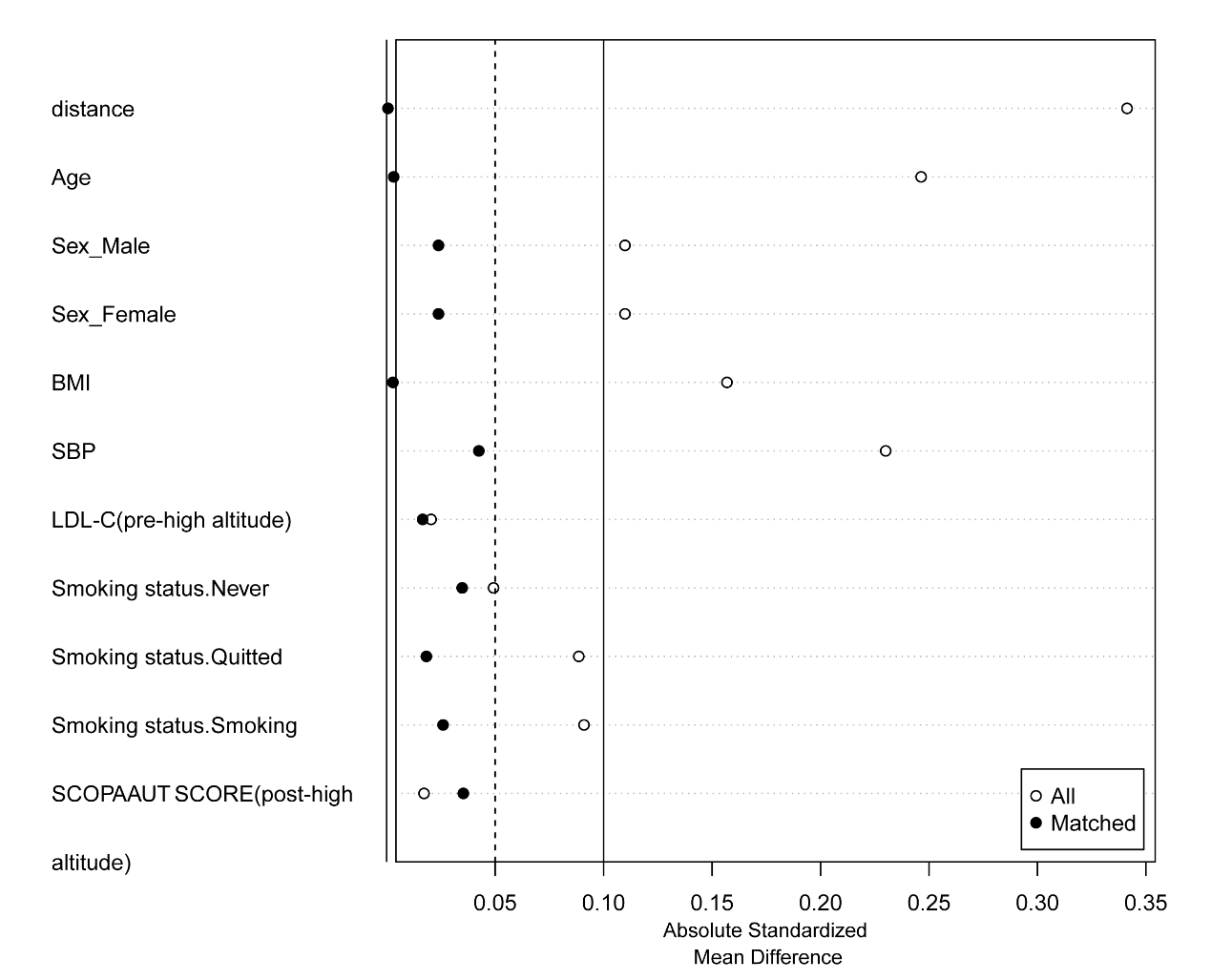


**Fig S2. Love Plot for Standardized Mean Differences in Covariates Before and After Propensity**

**Table S1. Covariate Balance After PSM.**

|  | **Means Treated** | **Means Control** | **Std. Mean Diff.** | **Var. Ratio** | **eCDF Mean** | **eCDF Max** | **Std. Pair Dist.** |
| --- | --- | --- | --- | --- | --- | --- | --- |
| distance | 0.09426961 | 0.09424304 | 0.0005892108 | 1.0038600 | 0.0003821048 | 0.008658009 | 0.002039227 |
| Age | 25.63636364 | 25.61904762 | 0.0032270752 | 0.9087385 | 0.0144751082 | 0.047619048 | 0.626052592 |
| Sex_Male | 0.97402597 | 0.97835498 | 0.0238725032 |  | 0.0043290043 | 0.004329004 | 0.214852529 |
| Sex_Female | 0.02597403 | 0.02164502 | 0.0238725032 |  | 0.0043290043 | 0.004329004 | 0.214852529 |
| BMI | 22.75238095 | 22.74588745 | 0.0028695750 | 0.9346678 | 0.0153544372 | 0.051948052 | 0.950977163 |
| SBP | 117.99567100 | 118.50216450 | 0.0424873443 | 1.0522596 | 0.0136577038 | 0.047619048 | 0.776029529 |
| LDL-C  (pre-high altitude) | 2.89346320 | 2.90064935 | 0.0165828992 | 0.8582442 | 0.0131845341 | 0.056277056 | 1.225136598 |
| Smoking status.Never | 0.44588745 | 0.46320346 | 0.0347991059 |  | 0.0173160173 | 0.017316017 | 0.835178542 |
| Smoking status.Quitted | 0.06060606 | 0.05627706 | 0.0182892429 |  | 0.0043290043 | 0.004329004 | 0.493809558 |
| Smoking status.Smoking | 0.49350649 | 0.48051948 | 0.0259799071 |  | 0.0129870130 | 0.012987013 | 0.822697058 |
| SCOPAAUT SCORE (post-high altitude) | 6.71861472 | 6.91341991 | 0.0353281065 | 1.2140042 | 0.0122850123 | 0.051948052 | 0.978981085 |

High altitude was defined as an altitude ≥2,500 m. BMI: body mass index; LDL-C: Low-Density Lipoprotein Cholesterol; SBP: systolic blood pressure. SCOPA-AUT: the Scale for Outcomes in Parkinson′s Disease for Autonomic Symptoms; PSM: Propensity Score Matching.
